# Significant impacts of the COVID-19 pandemic on race/ethnic differences in US mortality

**DOI:** 10.1073/pnas.2205813119

**Published:** 2022-08-23

**Authors:** José Manuel Aburto, Andrea M. Tilstra, Ginevra Floridi, Jennifer Beam Dowd

**Affiliations:** ^a^Leverhulme Centre for Demographic Science, Department of Sociology, and Nuffield College, University of Oxford, Oxford, OX1 1JD, United Kingdom;; ^b^Department of Population Health, London School of Hygiene and Tropical Medicine, London, WC1E 7HT, United Kingdom;; ^c^Interdisciplinary Centre on Population Dynamics, University of Southern Denmark; Odense 5000, Denmark;; ^d^University of Colorado Population Center, Institute of Behavioral Science, University of Colorado Boulder, Boulder, CO 80302

**Keywords:** COVID-19, demography, life expectancy, lifespan inequality, years of life lost

## Abstract

The coronavirus 2019 (COVID-19) pandemic triggered global declines in life expectancy. The United States was hit particularly hard among high-income countries. Early data from the United States showed that these losses varied greatly by race/ethnicity in 2020, with Hispanic and Black Americans suffering much larger losses in life expectancy compared with White people. We add to this research by examining trends in lifespan inequality, average years of life lost, and the contribution of specific causes of death and ages to race/ethnic life-expectancy disparities in the United States from 2010 to 2020. We find that life expectancy in 2020 fell more for Hispanic and Black males (4.5 and 3.6 y, respectively) compared with White males (1.5 y). These drops nearly eliminated the previous life-expectancy advantage for the Hispanic compared with the White population, while dramatically increasing the already large gap in life expectancy between Black and White people. While the drops in life expectancy for the Hispanic population were largely attributable to official COVID-19 deaths, Black Americans saw increases in cardiovascular diseases and “deaths of despair” over this period. In 2020, lifespan inequality increased slightly for Hispanic and White populations but decreased for Black people, reflecting the younger age pattern of COVID-19 deaths for Hispanic people. Overall, the mortality burden of the COVID-19 pandemic hit race/ethnic minorities particularly hard in the United States, underscoring the importance of the social determinants of health during a public health crisis.

## Significance

Public interest in social and health inequalities is increasing. We examine the impact of coronavirus 2019 (COVID-19) on mortality in the United States across racial/ethnic groups and present four key findings. All groups suffered sizable life-expectancy losses and increases in years of life lost in 2020. Mortality from cardiovascular diseases, “deaths of despair,” and COVID-19 explained most of these losses. Working-age mortality accounted for substantial life-expectancy losses, especially among Hispanic males. Lifespan inequality increased for Hispanic and White people but decreased slightly for Black people. Finally, the pandemic shifted racial/ethnic mortality differentials in favor of White people, narrowing the Hispanic advantage and widening the Black disadvantage. Our results provide a comprehensive assessment of mortality trends to inform policies targeting inequalities.

The coronavirus 2019 (COVID-19) pandemic has taken an unprecedented toll on mortality around the world. Most high-income countries experienced life-expectancy losses in 2020 (1–4), and many continued to experience declines in 2021 (5). The United States saw its largest drop in life expectancy (1.7 y for females and 2.1 y for males) in recent history (1), with COVID-19 deaths accounting for most of the decline for both females and males (6). Early data showed uneven impacts of the pandemic by race/ethnicity in the United States. The Center for Disease Control and Prevention estimates that between 2019 and 2020, life expectancy decreased by 3 y for the Hispanic population and by 2.9 y for the non-Hispanic Black (henceforth, Black) population, compared with a 1.2-y decline for non-Hispanic White (henceforth, White) people (6). The decrease was largest among Hispanic males (3.7 y), followed by Black males (3.3 y), and was smallest among White females (1.1 y). These findings are consistent with early studies projecting a disproportionate impact of the pandemic on life expectancy among race/ethnic minorities (7). It is important to monitor these disparities, which reflect underlying inequalities that are often magnified during a public health crisis (8). Both direct deaths from COVID-19 infection (9) as well as indirect deaths from other causes likely disproportionately affected race/ethnic minorities during the pandemic because of the social and economic disadvantages of historically marginalized populations in the United States (6, 10).

This study provides a comprehensive analysis of mortality changes across racial/ethnic groups in the United States before and during the first year of the pandemic. It contributes to the evidence by 1) analyzing recent trends in life expectancy, average years of life lost (AYLL), and lifespan inequality from 2010 to 2020 separately for Black, Hispanic, and White populations; 2) identifying the ages and causes of death driving recent changes, including deaths from cardiovascular diseases (CVDs), respiratory diseases, infectious and parasitic diseases, “deaths of despair” (i.e., suicide-, drug-, and alcohol-related mortality), cancers, accidents, and COVID-19; and 3) comparing race/ethnic gaps in these outcomes before and during the pandemic.

In the years before the pandemic, life expectancy in the United States followed atypical trends of stagnation that have not been observed in most high-income countries (6, 11, 12). These trends have been marked by worsening working-age mortality due to increased drug-related causes of death (13–17), as well as increased deaths from CVD at middle and later ages (18). Life expectancy is consistently higher for the White population relative to the Black population, although the gap between Black and White people narrowed from 5.7 y in 2000 to 3.8 y in 2010, (19). This convergence is partly due to relative improvements in mortality from heart diseases, HIV/AIDS, accidents, and cancer (20, 21). By contrast, the Hispanic population had higher life expectancy than the White population throughout the prepandemic period, attributable to lower mortality from cancer, CVD, diabetes, chronic respiratory diseases, perinatal conditions, as well as deaths of despair. Early evidence suggests that the pandemic has widened the White-Black gap in life expectancy, while reducing the Hispanic advantage (6). However, less is known about which ages and causes of death drove these changes (10).

The COVID-19 pandemic has directly and indirectly affected multiple causes of death. For example, delays in treatment may have increased mortality from cancers (22), or avoidance of hospitals for fear of infection may have increased mortality from acute cardiovascular events (23). COVID-19 is also associated with elevated risk of cardiovascular events and diabetes in the months following infection (24, 25). Crucially, the impact of these changes likely varies across race/ethnic groups, due to differences in socioeconomic resources, rates of health insurance, and access to health care (7). Recent findings show that, while COVID-19 death rates were highest in the Hispanic population, Black people experienced exceptionally large increases in mortality from heart disease, diabetes, and external causes of death (10).

So far, research on the differential impact of the pandemic on mortality across race/ethnic groups has mainly relied on estimates of overall life expectancy (6, 7, 26, 27) and standardized death rates (10). We extend these previous estimates by examining the ages and specific causes of death contributing to changes in life expectancy by race/ethnicity. While life expectancy is a widely used and important indicator for studying population health and mortality, it is an average measure that conceals population variability and inequality (28–31). Lifespan inequality captures a fundamental type of inequality: variation in length of life (32). Two populations that share the same life expectancy could experience differences in the variation around the timing of death. For instance, a high lifespan inequality measure would suggest that deaths occur within a wider age range, while a low measure of lifespan inequality would suggest a narrower age range. Hence, lifespan inequality, measured as the spread of ages at death in a population (e.g., SD), reflects how predictable length of life is at the individual level, and it underlies how uneven mortality improvements are at the population level (32, 33).

Black Americans not only experience shorter life expectancy compared with Hispanic and White people but also have less predictable lifespans, with higher lifespan inequality (34, 35). The impact of the pandemic on US lifespan inequality is currently unknown. Evidence from England and Wales shows that both lifespan inequality and life expectancy decreased during 2020 because mortality was concentrated at older ages (36). However, in the United States, life-expectancy losses during the pandemic have been driven by increases in mortality at both older and working ages (1, 5, 37). Previous studies show that increased midlife death rates increase lifespan inequality (38–40).

A complementary indicator to life expectancy is AYLL (41). This refers to the AYLL between birth and an upper age limit, often 95 y, from a synthetic cohort experiencing death rates in a given year throughout their lifespans. For example, if individuals between birth and age 95 live on average 80 y, then there are 15 y of life lost. While other indicators of years of life lost simply add up estimated remaining life expectancies among observed deaths (42, 43), AYLL is comparable across populations and over time, and is not affected by population age structure (44). Unlike life expectancy, this indicator enables researchers to quantify the burden of specific causes of death in a given year in a comparable way between populations (45), which is particularly important when comparing the impact of the pandemic across countries or groups with very different age structures. Conceptualized as such, AYLL represents a useful complement to life expectancy at birth, because it provides a snapshot of the contribution of different causes of death to years lost, rather than only how different causes contribute to changes in life expectancy over time. Using life expectancy, lifespan inequality, and AYLL, we comprehensively quantify the unequal impact of age- and cause-specific mortality before and during the first year of the pandemic across race/ethnic groups in the United States.

## Life Expectancy, Lifespan Inequality, and Years of Life Lost

**Fig. 1.**
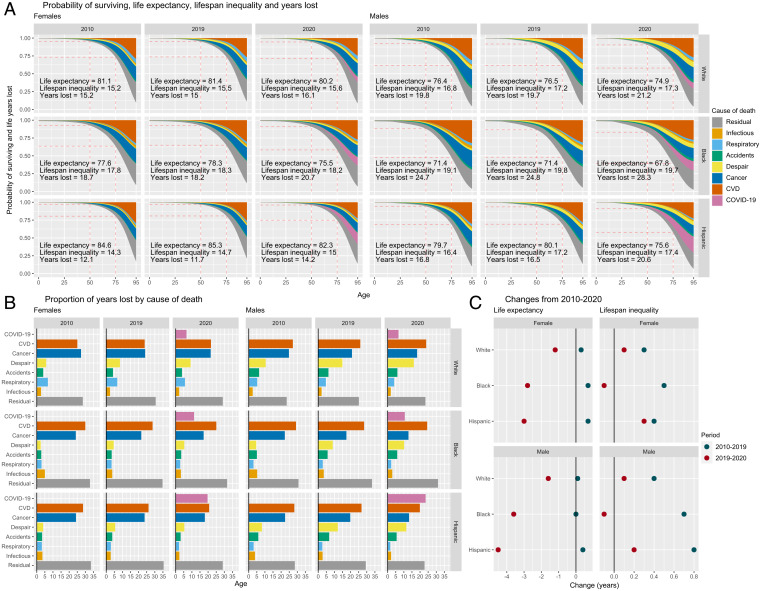
(*A*) Probability of surviving and AYLL by cause of death (colored areas, *Top*), life expectancy, lifespan inequality, and years of life lost by year, sex, and ethnic/racial group. (*B*) Proportion of life years lost by cause of death. (*C*) Changes in life-expectancy and lifespan inequality.

Life expectancy for both females and males stagnated in the second decade of the 21st century for all race/ethnic groups, but particularly for White people (Fig. 1*A*). Black and Hispanic females saw small improvements of only 6 mo from 2010 to 2019, from 77.6 to 78.3 y and from 84.6 to 85.3 y, respectively. Black males had the lowest life expectancy throughout the period—71.4 y in 2019. Lifespan inequality, measured by the SD of ages at death (as described in *Materials and Methods*), increased in 2010–2019 for all groups and was highest in the Black population. This means that the Black population faces a double mortality burden compared with White and Hispanic people, with both shorter *and* more unpredictable lives. AYLL in people younger than age 95 varied from 11.7 y for Hispanic females to 24.8 y for Black males, with little to no improvements over this period. In Fig. 1*A*,) AYLL is shown by the total colored area above the survival (probability of surviving) curve and can be attributed to specific causes of death. Deaths from CVD accounted for the largest individual share of life lost in 2010 and 2019 (between 25% and 30%) (Fig. 1*B*), followed by cancer (more than 20%). In 2019, deaths of despair accounted for 10+% of AYLL in males, with the biggest impact for White males.

In 2020, life expectancy decreased for all groups (Fig. 1*C*). Hispanic and Black males saw the largest drops (4.5 and 3.6 y, respectively), while their female counterparts experienced losses of almost 3 y. White females and males lost 1.2 and 1.5 y of life expectancy, respectively. Lifespan inequality increased slightly for Hispanic and White populations but decreased slightly for the Black population. Premature mortality at younger ages translates into increased lifespan inequality, but increases in mortality at older ages may decrease lifespan inequality. Hence, the divergent patterns in lifespan inequality across groups suggest the pandemic hit race/ethnic groups differently by age. AYLL also increased substantially for all groups. The largest increase in AYLL in 2020 was found in Hispanic males (4.1 y), where COVID-19 contributed the most among specific causes (almost 25% of life lost) (Fig. 1*B*), followed by Black males (3.5 y), where COVID-19 accounted for more than 10% of the total years lost.

## Trends in Life Expectancy and Lifespan Inequality by Age and Causes of Death

**Fig. 2.**
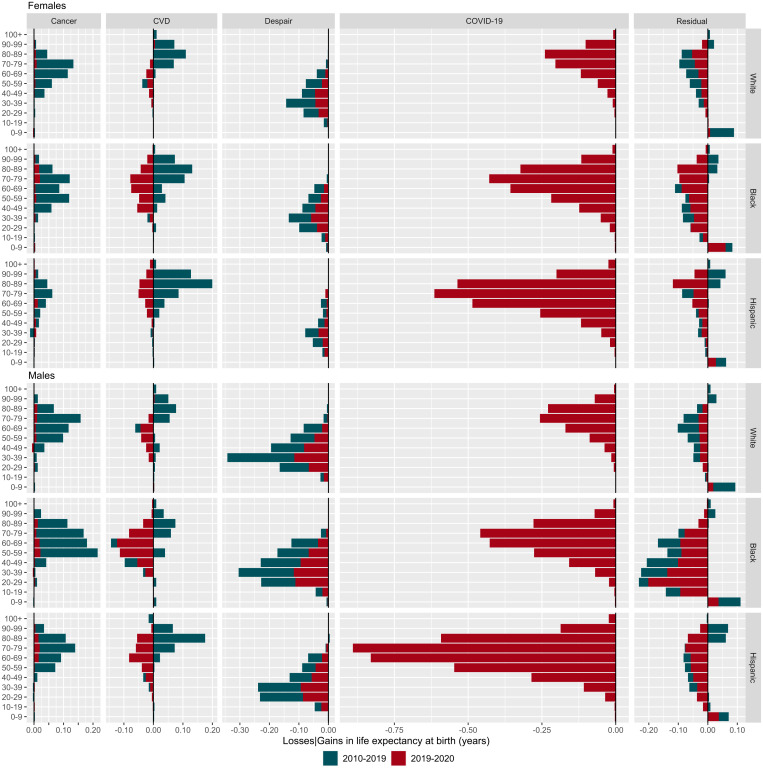
Contributions by age and causes of death to changes in life expectancy in 2010–2019 and 2019–2020 by race/ethnic groups and sex (*SI Appendix*, Fig. S1 shows infectious and respiratory diseases).

The stagnation of life expectancy in the United States from 2010 to 2019 reflected changing age patterns of mortality in certain causes of death. Improvements in cancer mortality, predominantly at ages 50–90, contributed to increases in life expectancy across all groups in this period (Fig. 2) (*SI Appendix*, Fig. S1 shows infectious and respiratory diseases). The biggest gains from cancer improvements were observed for Black females and males (0.42 and 0.69 y), and White males (0.48 y) (*SI Appendix*, Fig. S2). Improvements in cardiovascular mortality also contributed positively to life expectancy in 2010–2019, especially for females (0.24 y for White, 0.40 y for Black, and 0.47 y for Hispanic females). However, this progress was reversed by increased mortality from deaths of despair, with males in all race/ethnic groups losing a staggering half a year or more from deaths of despair from 2010 to 2019. Losses from deaths of despair were concentrated at younger ages, with 30- to 39-year-olds suffering the greatest life-expectancy losses among all race/ethnic groups. While females also lost life expectancy from deaths of despair in 2010–2019, the magnitude was smaller compared with males (0.16 y).

The pandemic induced changes in the patterns of mortality by cause of death (Fig. 2, red color). In 2020, small cancer improvements translated into negligible gains in life expectancy, while deaths from cardiovascular mortality, deaths of despair, and residual mortality increased dramatically. These changes translated into significant overall losses of life expectancy. For example, deaths of despair contributed to life-expectancy declines of 0.35 y for White, 0.46 y for Black, and 0.33 y for Hispanic males from 2019 to 2020, on top of already persistently high levels of deaths of despair in the previous decade. As expected, the largest contributions to life expectancy losses were from deaths due to COVID-19. Official COVID-19 deaths translated into losses of 0.88 y for White, 1.78 y for Black, and 3.50 y for Hispanic males. The corresponding losses among females were 0.78, 1.65, and 2.31 y. In contrast to other high-income countries, in the United States, COVID-19 deaths at working ages contributed significantly to life-expectancy declines. For example, among Hispanic and Black males, mortality below age 60 accounted for 29.9% and 31.9% of the total decline due to COVID-19 in life expectancy, compared with 18.4% for White people. For females, the corresponding proportions were 14.7%, 27.2%, and 21.0% for White, Black, and Hispanic people, respectively.

Increased mortality at any age translates into life-expectancy losses. However, for lifespan inequality to increase, mortality deterioration needs to be concentrated in younger ages. This means that worsening mortality at older ages, above a threshold age usually very close to life expectancy, may lead to decreased lifespan inequality (46–48). For example, lifespan inequality decreased in England and Wales in 2020 due to worsening death rates at older ages during the pandemic. Causes of death that explain increases or decreases in life expectancy are often not the same driving decreases or increases in lifespan inequality (49, 50). In the United States, lifespan inequality increased for all groups between 2010 and 2019. Increases were largely driven by mortality from deaths of despair below age 50 and improvements in CVD mortality at ages 70+ (*SI Appendix*, Fig. S3). In 2020, lifespan inequality continued to increase for White and Hispanic people, while the Black population experienced small declines. Increased mortality at older ages from COVID-19 contributed to decreasing lifespan inequality among Black and White people. Among Hispanic males, COVID-19 deaths contributed to increasing lifespan inequality because they were concentrated at younger ages, increasing uncertainty in lifespan. Deaths of despair continued to increase lifespan inequality in 2020 for all groups.

## Life-Expectancy and Lifespan Inequality Gaps between Race/Ethnic Groups

**Fig. 3.**
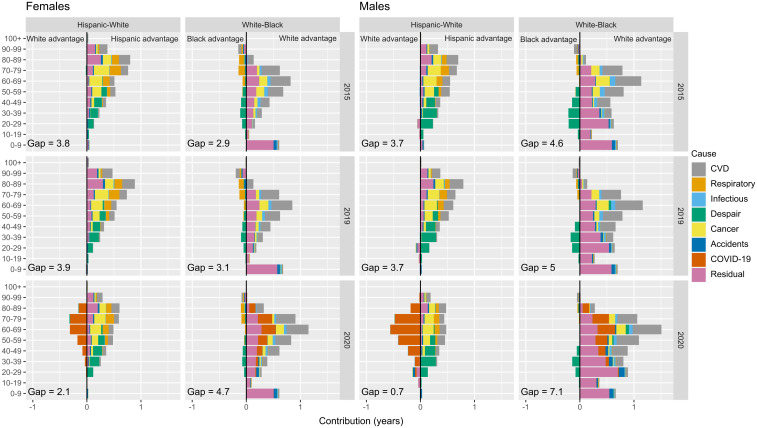
Contributions by age and causes of death to race/ethnic gaps in life expectancy in 2015, 2019, and 2020 by sex.

Hispanic people have consistently had the highest life expectancy among all race/ethnic groups in the United States, and Black people had the lowest. Between 2015 and 2019, life-expectancy gaps between the Hispanic and White populations remained stable at around 3.8 y for both females and males (Fig. 3). The gap between White and Black males increased from 4.6 to 5 y in 2015–2019, while for females it increased minimally from 2.9 to 3.1 y. Fig. 3 shows age- and cause-specific contributions to the gap in Hispanic-White and White-Black life expectancy in 2015, 2019, and 2020 (Hispanic-Black comparisons described in *SI Appendix*, Fig. S4). In 2015 and 2019, the Hispanic-White gap was largely explained by a Hispanic advantage in mortality at adult ages (20 and older) for both females and males. In these years, the causes of death that contributed the most to the gap were cancer, deaths of despair, CVDs, and residual mortality (*SI Appendix*, Fig. S5). In contrast, higher mortality under age 10 among Black people contributed substantially to the White-Black gap in life expectancy. Deaths from CVD and residual causes contributed the most to the White-Black gap. The only causes of death less severe in Black compared with White people were deaths of despair at younger adult ages (20–59) and respiratory diseases at older ages (60+), both of which show negative contributions in Fig. 3. This means that if Black people were to experience the same mortality rates from deaths of despair and respiratory diseases as White people, the gap would be even larger.

In 2020, over the course of the pandemic, the Hispanic-White life-expectancy gap narrowed to 2.1 y for females and 0.7 y for males, a decline of 1.7 and 3.1 y, respectively. Conversely, the White-Black gap widened to 4.7 y for females and 7.1 y for males, an increase of 1.6 and 2.1 y, respectively. The decrease in the Hispanic-White gap was driven by the disproportionate impact of COVID-19 deaths at working and older ages among Hispanic people and does not reflect improvements in White mortality (negative values in Fig. 3). The White disadvantage accounting for the gap was evident at ages below 60 from deaths of despair and from respiratory diseases and cancer at older ages. The increasing White-Black gap in 2020 was largely explained by increased COVID-19 mortality above age 40, together with cardiovascular mortality. Other non-COVID cause-specific contributions to the White-Black gap remained similar to previous years, but it is worth noting that the Black “advantage” in deaths of despair had gotten smaller during the COVID-19 pandemic in 2020. These trends in mortality also affected lifespan inequality (*SI Appendix*, Fig. S6), but to a lesser extent. In 2020, the Hispanic population had the lowest lifespan inequality, and the Black population had the highest.

## Outlook and Implications

Previous pandemics, such as the 1918 Spanish influenza and the 2009 H1N1 influenza, affected population health and mortality differently across subpopulations (51). Overwhelming evidence, including our own, shows that so far, the COVID-19 pandemic has disproportionately affected racial/ethnic minorities in the United States (6, 7, 10, 52). We have provided a comprehensive analysis of mortality profiles for the White, Black, and Hispanic populations by age and cause of death in 2010–2019 and 2020, using a set of complementary demographic indicators of average mortality and its variability. Our findings show that the pandemic substantially affected existing mortality differentials in the United States in favor of the White population, narrowing the Hispanic advantage and widening the Black disadvantage in life expectancy and related measures. Comparing changes in 2020 with the previous decade puts into sharp relief the magnitude of mortality increases during the pandemic. As shown in Fig. 2, all racial/ethnic groups in the United States witnessed a decade of little change (2010–2019) followed by a year of dramatic increases in cause-and age-specific mortality. Moreover, the gaps in life expectancy between the groups also changed dramatically (Fig. 3), following a long period of relative stability in the Hispanic advantage and Black disadvantage compared with White people.

One year of pandemic-associated mortality nearly eliminated the Hispanic life-expectancy advantage that was consistent over the 2010–2019 period. The declining Hispanic mortality advantage was predominantly driven by a greater concentration of COVID-19 deaths among working-age Hispanic people compared with the White population, a finding that has also been documented elsewhere (6, 10). Given that the Hispanic population is much younger than the White population, the concentration of deaths among working-age Hispanic people indicates a disproportionate overall burden of the pandemic on this group in terms of lives lost to COVID-19, which is masked by the underlying assumptions of life-expectancy estimates. This is bolstered by our finding that lifespan inequality increased for the Hispanic population, indicating greater variability in ages at death among Hispanic people in the United States. These patterns can be attributed to a higher likelihood of viral exposure (due to employment and housing) and lower access to health care (52). The high burden of COVID-19 mortality in the Hispanic population, especially at working ages, suggests that exposure-related social factors outweighed any protection from previously accumulated health advantages. A key question is whether the Hispanic population will recover its life-expectancy advantage in the short, medium, or long term despite its socioeconomic disadvantage relative to the White population (the so-called Hispanic paradox) (53). Given that the reduction in the Hispanic-White life expectancy gap was largely explained by direct deaths due to COVID-19, vaccination rollout, and age-specific uptake may be most relevant in the short term as exposure-related differences become less salient. Recent vaccination data suggest that Hispanic people are slightly more likely than White people to have received at least one dose (64% vs. 62%) (54).

The ability to socially distance and minimize exposure to the virus varied greatly between those able and those unable to work remotely. Essential workers were exempted from “stay-at-home” orders in most states and were crucial to providing food and other vital services. Black and Hispanic people were much more likely than White people to work in jobs placing them at higher risk of exposure to the virus (52). Relatedly, unlike European countries, US COVID-19 policy responses were focused on the provision of unemployment benefits rather than wage compensation schemes (55). Individuals from racial/ethnic minorities may have been disproportionately likely to continue working due to their lower ability to access unemployment benefits (e.g., because of no residence permit for non-US citizens or more limited access to information on benefits for non-English speakers). While an assessment of the unequal effect of policy responses to the pandemic is beyond the scope of this study, the differences in the probability of dying from COVID-19 across racial/ethnic groups clearly highlight the need for policy responses to consider their potential for exacerbating existing inequalities.

In 2020, the persistent White-Black gap in life expectancy widened. This is the result of larger increases in COVID-19 and CVD mortality among Black people at older ages compared with their White peers, consistent with other studies (10). The increase is dramatic and warrants further investigation. Black people in the United States may have experienced a “syndemic” of COVID-19 and other chronic diseases (51). Our results suggest that, distinct from the Hispanic population, Black people may have suffered more from both direct COVID-19 deaths and indirect effects such as delayed or missed health care (10, 27). In contrast to the Hispanic-White gap, the increased White-Black gap in life expectancy may persist, due in part to lower vaccination rates among Black people relative to both White and Hispanic people (57% with at least one dose according to reference (54)). The gap could continue to widen in 2022 because of the proportional increase in the hospitalization of unvaccinated Black adults during the Omicron period (since December 2021) compared with other race/ethnic populations in the United States (56). Any indirect impacts of the pandemic that led to increases in CVD mortality may also persist longer than acute COVID-19 risks.

Somewhat paradoxically, lifespan inequality decreased slightly for Black people, because the increase in mortality was primarily at older ages. A similar result was documented in England and Wales for 2020 (36). Typically, life expectancy increases are accompanied by decreases in lifespan inequality (28, 30, 31). This means that countries that have achieved higher levels of life expectancy benefit from lower lifespan inequality. However, recent evidence has challenged the uniformity of this association. For example, in Eastern and Central Europe, life-expectancy and lifespan inequality moved independently from one another before the dissolution of the Soviet Union due to worsening mortality at younger ages related to alcohol consumption and improvements in mortality at older ages (57). Similarly, groups with lower socioeconomic status in Finland and Spain experienced life-expectancy increases and widening lifespan inequalities simultaneously usually due to high midlife mortality (32, 58). Our results are further evidence of the potential decoupling of life expectancy and lifespan inequality in the context of the pandemic, pointing to the importance of the age profile of mortality during a crisis for changes to the predictability of lifespan. Substantively, the decreasing lifespan inequality in the Black population implies that the pandemic made lifespans more concentrated by shifting some deaths that would have occurred at even older ages closer to the average life expectancy. By contrast, the pandemic contributed to making average lifespans less predictable and more unequal within the Hispanic and White populations due to significant losses at younger ages.

The impact of the pandemic may be particularly unequal in countries characterized by high social inequality (e.g., as measured by the Gini coefficient), such as the United States (51). Evidence from England—a comparatively unequal high-income country—suggests that South Asian and Black people had considerably higher risk of COVID-19 infection, hospitalization, and death than the White population (59, 60). More research aimed at understanding the link between social factors and racial/ethnic differences in the impact of the pandemic is needed. It is also important to expand this research to low- and middle-income countries, where the impact of the pandemic may have been particularly unequal due to a combination of high inequality and less developed health care systems.

In the United States, prospects for 2021 are pessimistic, and inequalities may have worsened. Recent evidence shows that in 2021 life expectancy continued to decrease in the United States, with an estimated further drop of 0.3 mo on top of 2020 losses (5). Relative to 2020, 2021 was also characterized by a shift to younger ages at death for COVID-19 for all racial/ethnic groups (61, 62). Racial/ethnic disparities in life-expectancy reductions due to COVID-19 persisted but were smaller than in 2020, with Black and Hispanic people experiencing reductions 1.3–1.9 times those of White people. Some evidence suggests higher COVID-19 mortality rates at ages below 85 for ethnic minorities compared with White people (61), while a recent report found that, in 2021, White people experienced life-expectancy declines but racial/ethnic minority groups did not (63). As such, 2021 trends are unclear and necessitate further examination once better data become available.

There are some limitations to this study related to the availability and quality of the data that were analyzed. It is possible that deaths due to COVID-19 were attributed to CVDs, and this misclassification may have disproportionately affected the Black population, driving the increase in CVD deaths in this group (10). However, empirical evidence on the mechanisms behind differential misclassification of deaths by racial/ethnic group is not yet clear. On the one hand, some studies suggest that vulnerable young adults from racial/ethnic minorities were the most likely to die outside of the hospital or in emergency departments, leading to systematic underreporting of COVID-19 deaths in these groups (64, 65). On the other hand, no differences across racial/ethnic groups emerge when comparing the ratio of non-COVID-19 mortality increases with COVID-19 increases between the first and the second half of 2020 (10). Assuming that misreporting was higher in the first 6 mo of 2020, this provides scant evidence that faulty cause-of-death assignments were significantly higher among Black people (10). Finally, because of data limitations, this study examines three race/ethnic groups and does not consider other minorities, including American Indians and Alaska natives, Asians, and other race/ethnic groups. For a more complete assessment of changes in mortality by racial/ethnic group during 2020, our results can be interpreted in conjunction with estimates of life-expectancy decline among Native Americans. These suggest a loss in life expectancy for Native Americans by 2.5 y in 2020, which is larger than the loss experienced by Black and White people (66).

Despite these limitations, this study offers several important lessons for scientists and policymakers. The pandemic has affected mortality unequally. COVID-19 mortality hit Black and Hispanic Americans especially hard, exacerbating existing White-Black disparities and nearly eliminating the Hispanic mortality advantage. Our findings of higher Hispanic mortality at working ages are consistent with previous work, suggesting increased risk of COVID-19 exposure for this group due to working conditions, housing, and access to preventive information (27, 52). The widening of the White-Black gap in life expectancy is particularly concerning because the difference between Black and White mortality prepandemic was already larger than the overall impact of the COVID-19 pandemic on White mortality (8).

Overall, our findings confirm the wide-ranging impact of the COVID-19 crisis on racial/ethnic differences in mortality in the United States. While the severe acute respiratory syndrome coronavirus 2 itself does not discriminate, the social environment shapes risk of infection and death in ways that reflect historical inequalities. Future work should continue to examine the direct impact of COVID-19 but also the impact of pandemic social and economic disruptions on racial/ethnic differences in health. Besides risk of increased socioeconomic disadvantage during the pandemic, Hispanic and Black people also suffered much higher levels of bereavement and loss from COVID-19 in their own families (67), contributing to significant trauma and stress that may have long-term health effects. Our study provides a comprehensive assessment of the impact of the COVID-19 pandemic on racial/ethnic gaps in mortality to ultimately inform research and policy interventions targeting such inequalities.

## Materials and Methods

### Data.

We used data from the publically available US multiple cause of death files, from the National Vital Statistics System division of the National Center for Health Statistics (https://www.cdc.gov/nchs/data_access/vitalstatsonline.htm#Mortality_Multiple) (68), and yearly population estimates compiled by the Surveillance, Epidemiology, and End Results Program (https://seer.cancer.gov/popdata/download.html) (69). Data are restricted to US residents in the years 2010–2020. Non-Hispanic White (White), non-Hispanic Black (Black), and Hispanic/Latino (Hispanic) populations were analyzed, and all other race/ethnic groups were excluded because of small data counts. We coded seven causes of death: CVD, respiratory diseases, infectious and parasitic diseases, deaths of despair, cancers, accidents (excluding those from deaths of despair), and COVID-19. While deaths of despair encompass three causes of death, driven by underlying phenomena that are likely independent (13, 14), evidence suggests that all three causes were rising before and during the pandemic. As such, we combined them here. Specific International Classification of Disease-10 codes are listed in Table 1.

**Table 1.** ICD codes for groups of causes of death

Category	ICD-10 Codes
CVD	I00-I78
Respiratory illness	J40-J46
Infectious and parasitic diseases	A00-A09, A16-A44, A48-A99, B00-B09, B15-B99, D86.9, G02, G14, H32, I32, I39 J17, K90.8, L44.4, L94.6, M02.3, M35.2, M66, N34.1, R11.1
Despair	F10-F16, F19, K70, K73-K74, U03, X40-X45, X64-X85, Y10-Y15, Y87
Cancer	C00-C99
Accidents	V00-V99, W00-W99, X00-X59, Y85-Y86 (excluding, despair causes)
COVID	U07.1, U07.2
Residual	All else

ICD-10: International Classification of Disease-10

### Life Table Construction.

Life tables for all race/ethnic groups by sex for the period 2010–2020 were constructed following standard demographic techniques consistent with a piece-wise constant hazard model using all-cause mortality by single ages, with the last age interval grouping deaths at ages 100+ (70).

### Life-Expectancy, AYLL, and Lifespan Inequality.

From the life tables, we retrieved life expectancy at birth and life years lost, and calculated lifespan inequality as an indicator of how spread out ages at death were. Life expectancy at birth is a summary indicator of population health and mortality. It expresses the average number of years a synthetic cohort of newborns is expected to live if they were to experience the mortality rates observed in a given year (71). It is not affected by population size or age structure, which makes it a preferred indicator for comparisons over time and between populations. At the same time, we should highlight that since life expectancy, by design, is not sensitive to differences in population size and age structure, this indicator does not allow us to easily compare overall burden of deaths in different groups (e.g., the disproportionate impact of the pandemic at younger ages could be considered even worse for Hispanic people because a greater share of their population is young).

AYLL is a complementary indicator of life expectancy. It refers to the average years lost of a synthetic cohort due to mortality in specific age groups or causes of death (41). For example, if individuals live on average 80 y between birth and age 95, then AYLL equals 15 y. In this analysis, we set the upper limit of age 95 to quantify AYLL. This strategy has been used previously to characterize mortality in contexts where subpopulations experience different mortality profiles, for example, populations with and without mental health disorders (44).

Lifespan inequality refers to variation in ages at death and is a marker of heterogeneity in mortality at the population level, and reflects uncertainty in lifetimes at the individual level (32). The greater lifespan inequality is, the more unpredictable lifespans are in a population. We measured lifespan inequality with the SD of the age-at-death distributions. Several indicators exist to measure lifespan inequality, including the life table entropy, the Gini coefficient, and life disparity, and all are highly correlated when measured from birth (72), which suggests that our results would not change on the basis of the indicator chosen.

### Decomposition of Life Expectancy by Age and Cause of Death.

In order to disentangle age- and cause-specific effects over time and between groups for life expectancy and lifespan inequality, we used the linear integral decomposition method (73), a state-of-the-art method that allows us to decompose the difference of two values of life expectancy or lifespan inequality by age and cause of death, which has been implemented previously for this type of analysis (e.g., (1)). Fig. 2 shows contributions by age and causes of death to changes in life expectancy in 2010–2019 and 2019–2020 by race/ethnic groups and sex. The bars in Fig. 2 are stacked, with blue colors depicting the age- and cause-specific contributions to the change in life expectancy between 2020 and 2019; while red colors refer to the change in life expectancy between 2019 and 2020 for each sex-ethnic/race group. In order to obtain these contributions, we decomposed each yearly change by age and cause of death using the linear integral decomposition method, and subsequently added them up to represent both periods 2010–2019 and 2019–2020. This means that the bars are additive for each sex-ethnic/race group. If, for example, we were to sum all the bars by age and cause of death (including both colors) for White females, we would obtain the total change in life expectancy between 2010–2020 for this group (i.e., from 81.1 to 80.2 y) *SI Appendix*, Fig. S4 shows the corresponding graph with nonstacked bars.

## Supplementary Material

Supplementary File

## Data Availability

The replication files for this paper include customized functionality written in the R statistical programming language (74). The code and all harmonized input and output data pertaining to our analysis are hosted on both Zenodo https://zenodo.org/record/6402403 (a general-purpose open-access repository developed under the European OpenAIRE program and operated by the European Council for Nuclear Research [CERN]) (75), and GitHub (https://github.com/jmaburto/ex_USA_racial-ethnic_differences) (76). Anonymized age- and cause-specific mortality data have also been deposited in Zenodo at jmaburto/ex_USA_racial-ethnic_differences: v1.0.0 (10.5281/zenodo.6402403) (75). All study data are included in the article and/or supporting information.
