# Supplementary material for: Significant impacts of the COVID-19 pandemic on race/ethnic differences in US mortality
Source: Proc Natl Acad Sci U S A. 2022 Aug 23;119(35):e2205813119. doi: 10.1073/pnas.2205813119 (PMC9436308; doi:10.1073/pnas.2205813119)

**Supplementary material for the paper: Significant impacts of the COVID-19 pandemic on race/ethnic differences in USA mortality**

**Authors:** José Manuel Aburto[1,2,3,*] Andrea M. Tilstra[1,2,4]*, Ginevra Floridi[1,2], Jennifer B. Dowd[1,2]

**Affiliations:**
[1]Leverhulme Centre for Demographic Science and Department of Sociology, University of Oxford; 42-43 Park End Street, Oxford, OX1 1JD, UK.
[2]Nuffield College; New Rd, Oxford, OX1 1NF, UK.
[3]Interdisciplinary Centre on Population Dynamics, University of Southern Denmark; Odense 5000, Denmark.
[4] University of Colorado Population Center, Institute of Behavioral Science, University of Colorado Boulder, Boulder, CO, USA
*Corresponding authors: JMA (jose-manuel.aburto@sociology.ox.ac.uk), AMT (andrea.tilstra@sociology.ox.ac.uk)

**Classification**: Social sciences, Demography

**Keywords:** COVID-19; demography; life expectancy; lifesan inequality; years of life lost

**Supplementary Information**

Figure S1.*Contributions by age groups and causes of death to changes in life expectancy in 2010-2019 and 2019-2020 by racial/ethnic groups and sex.*

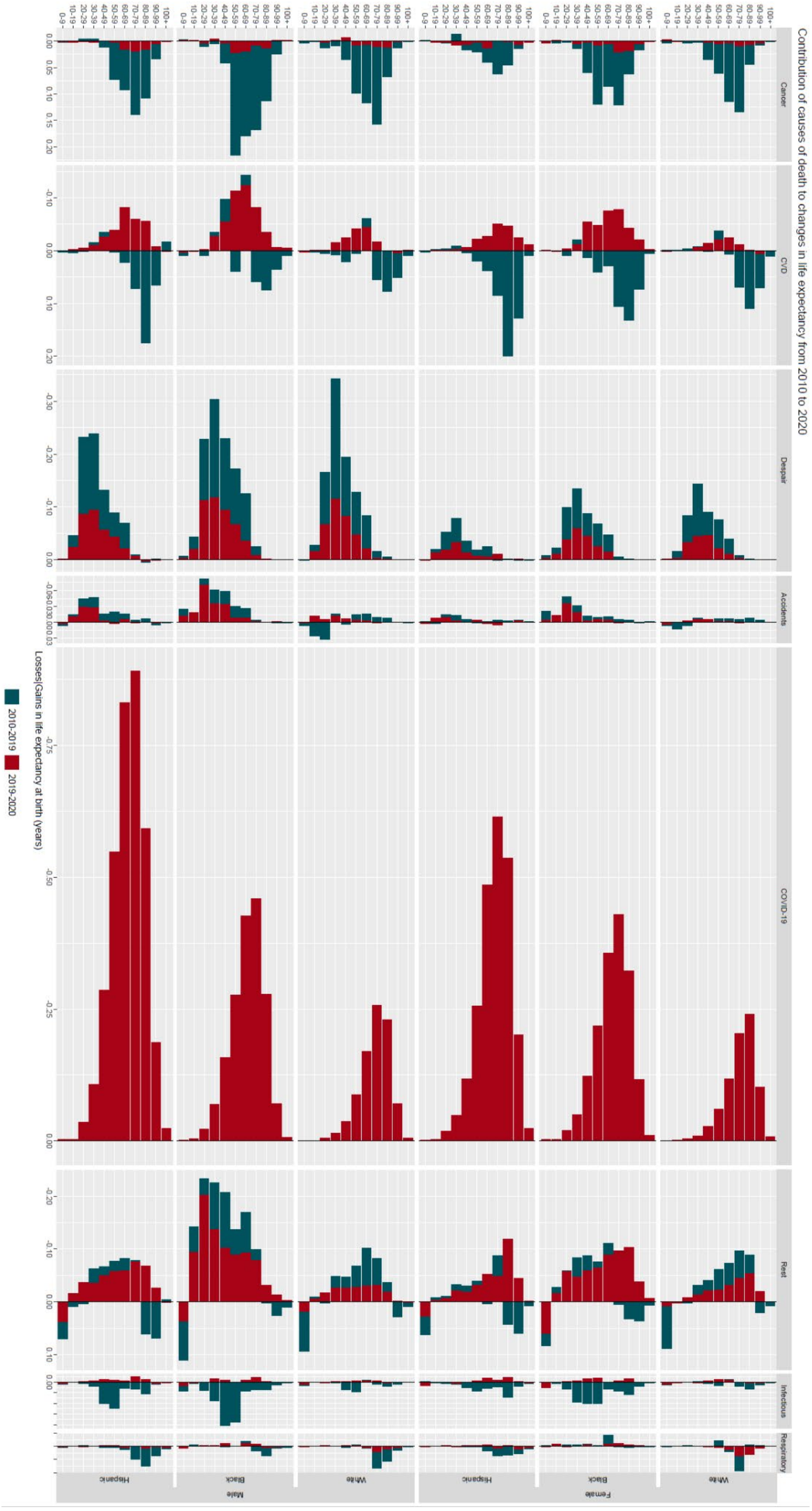

*Figure S2. Contributions by causes of death to changes in life expectancy in 2010-2019 and 2019-2020 by racial/ethnic groups and sex.*

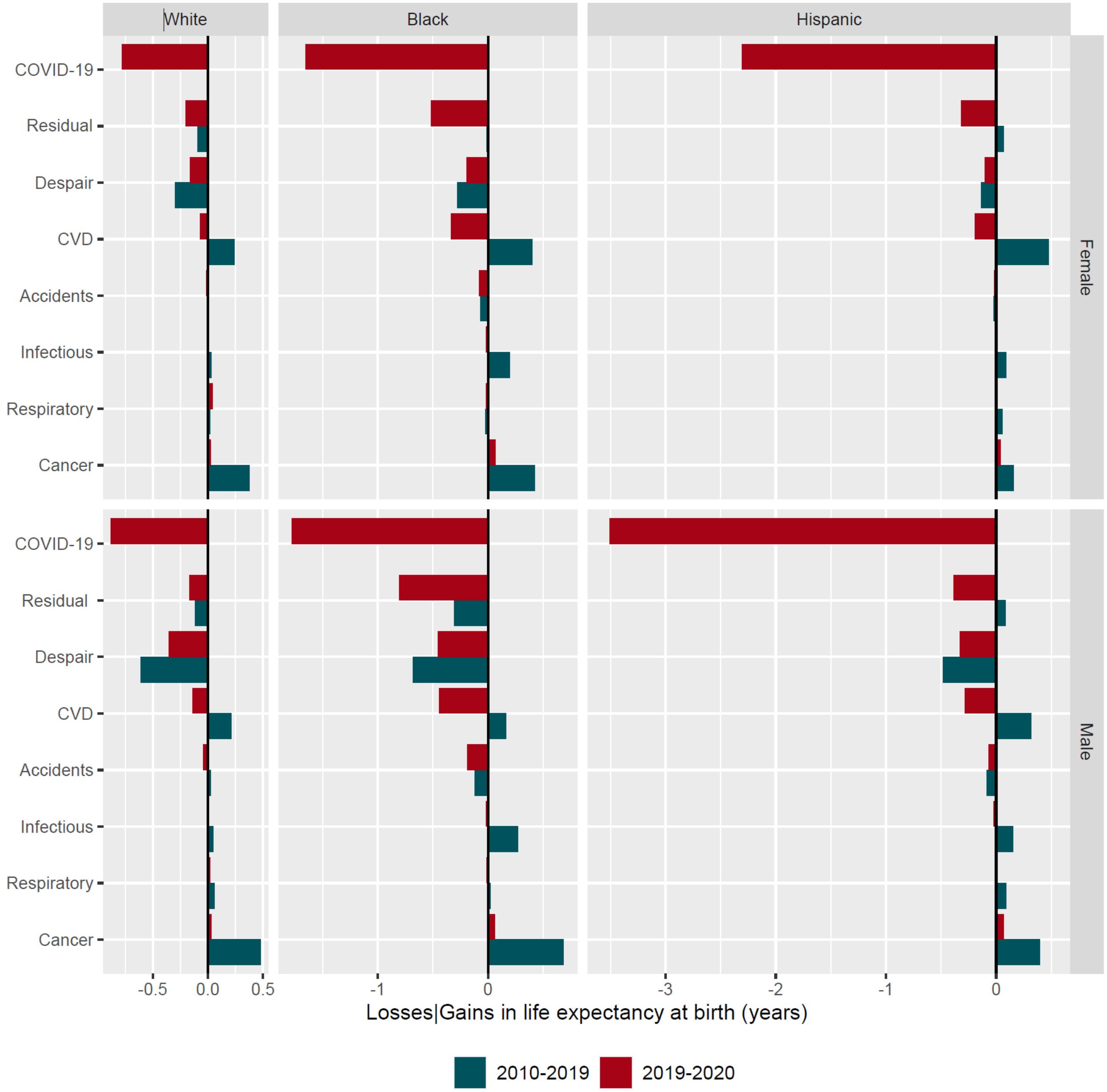

*Figure S3. Contributions by causes of death to changes in lifespan inequality in 2010-2019 and 2019-2020 by racial/ethnic groups and sex.*

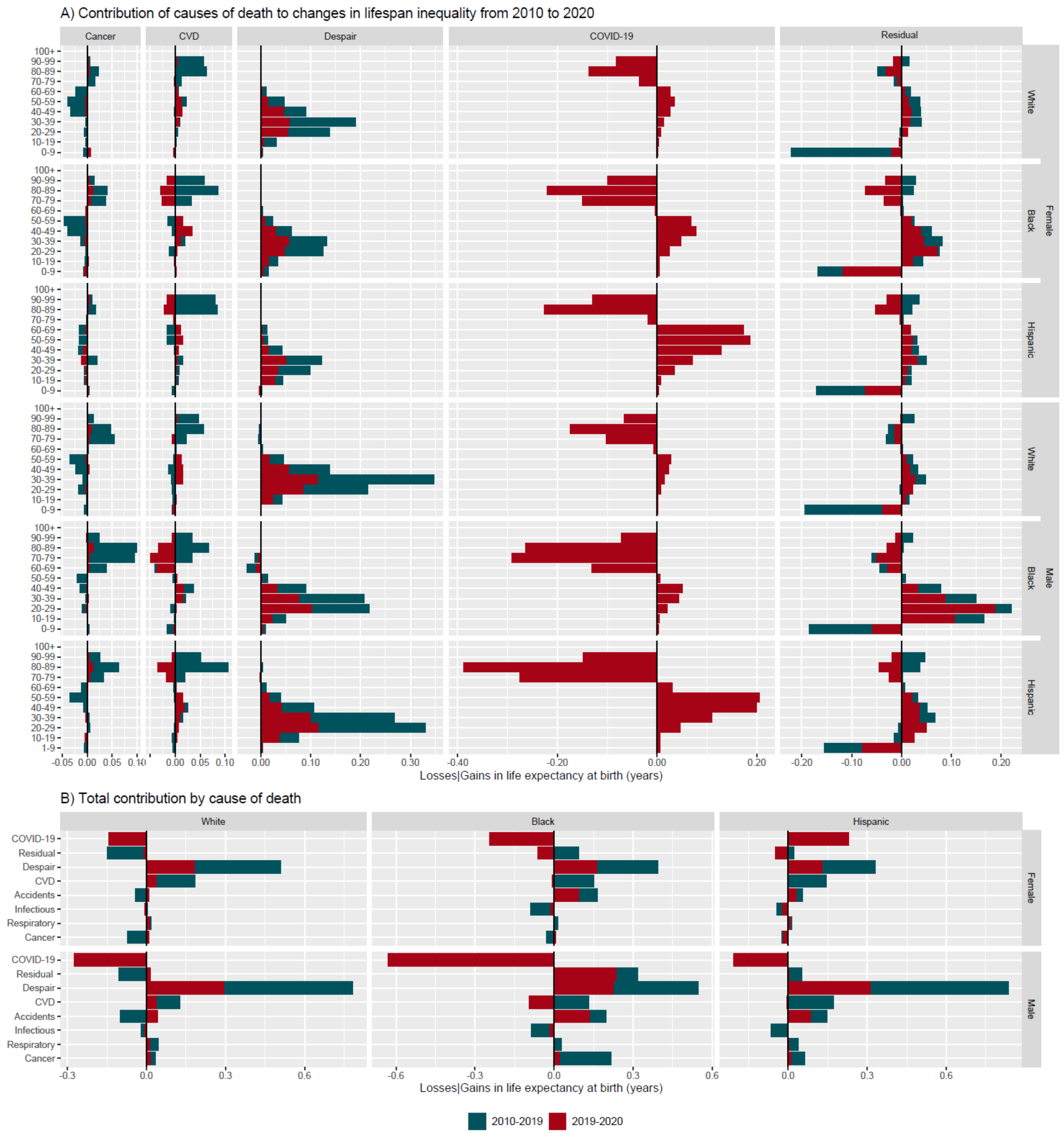

Figure S4. *Results as shown in Figure 3 for Hispanic-Black difference in life expectancy.*

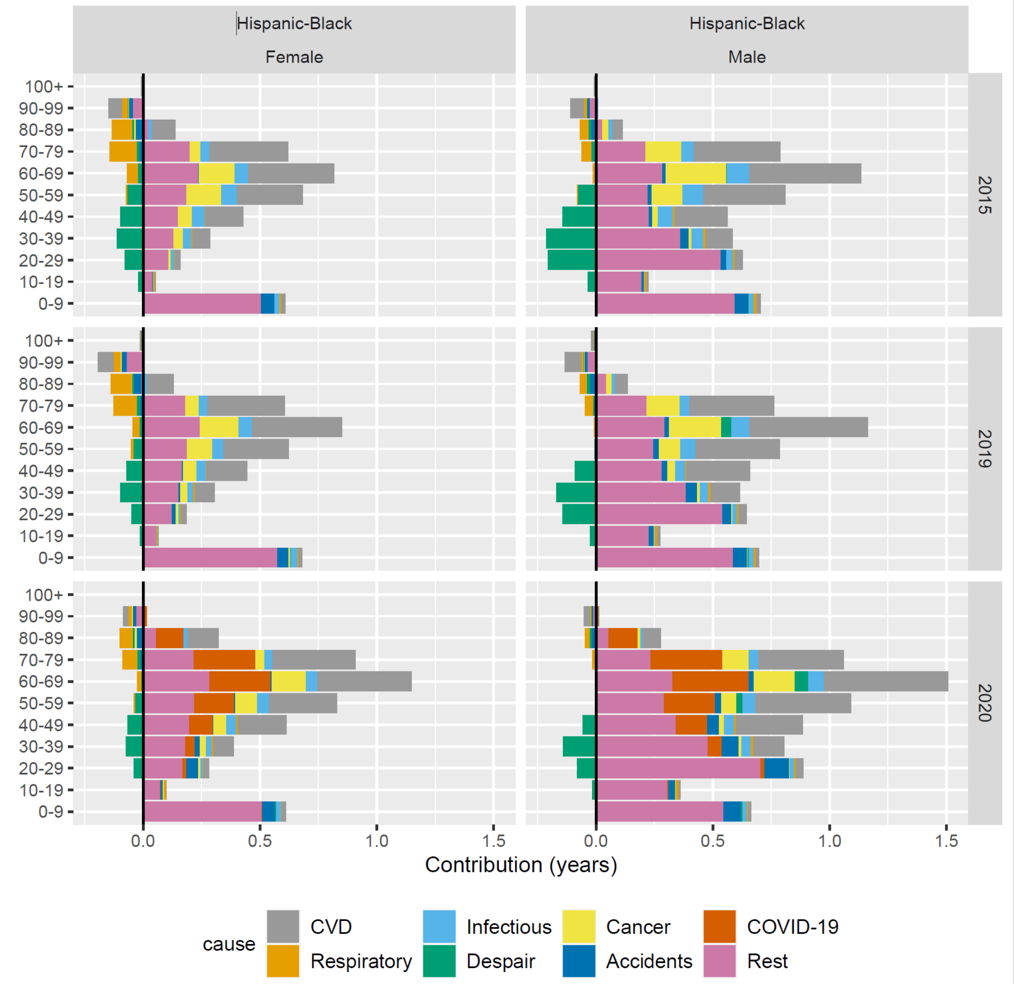

Figure S5. *Contributions by causes of death to ethnic/racial gaps in life expectancy in 2015, 2019 and 2020 by sex*

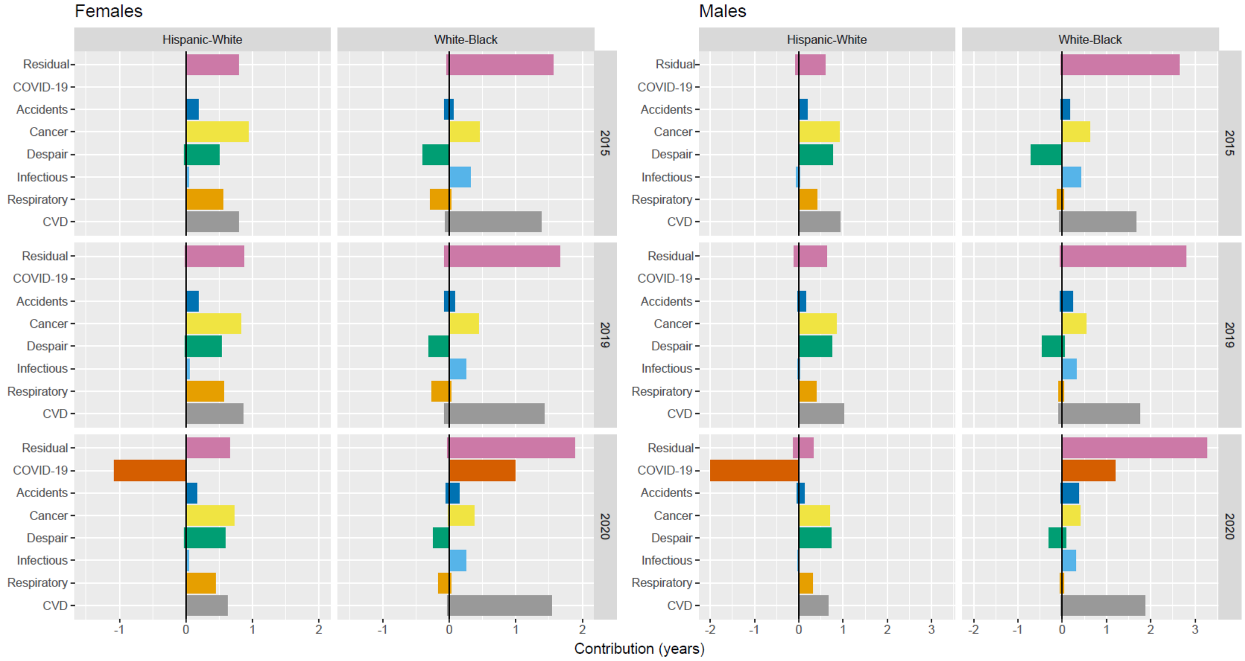

Figure S6 *Contributions by age and causes of death to ethnic/racial gaps in lifespan inequality in 2015, 2019 and 2020 by sex*

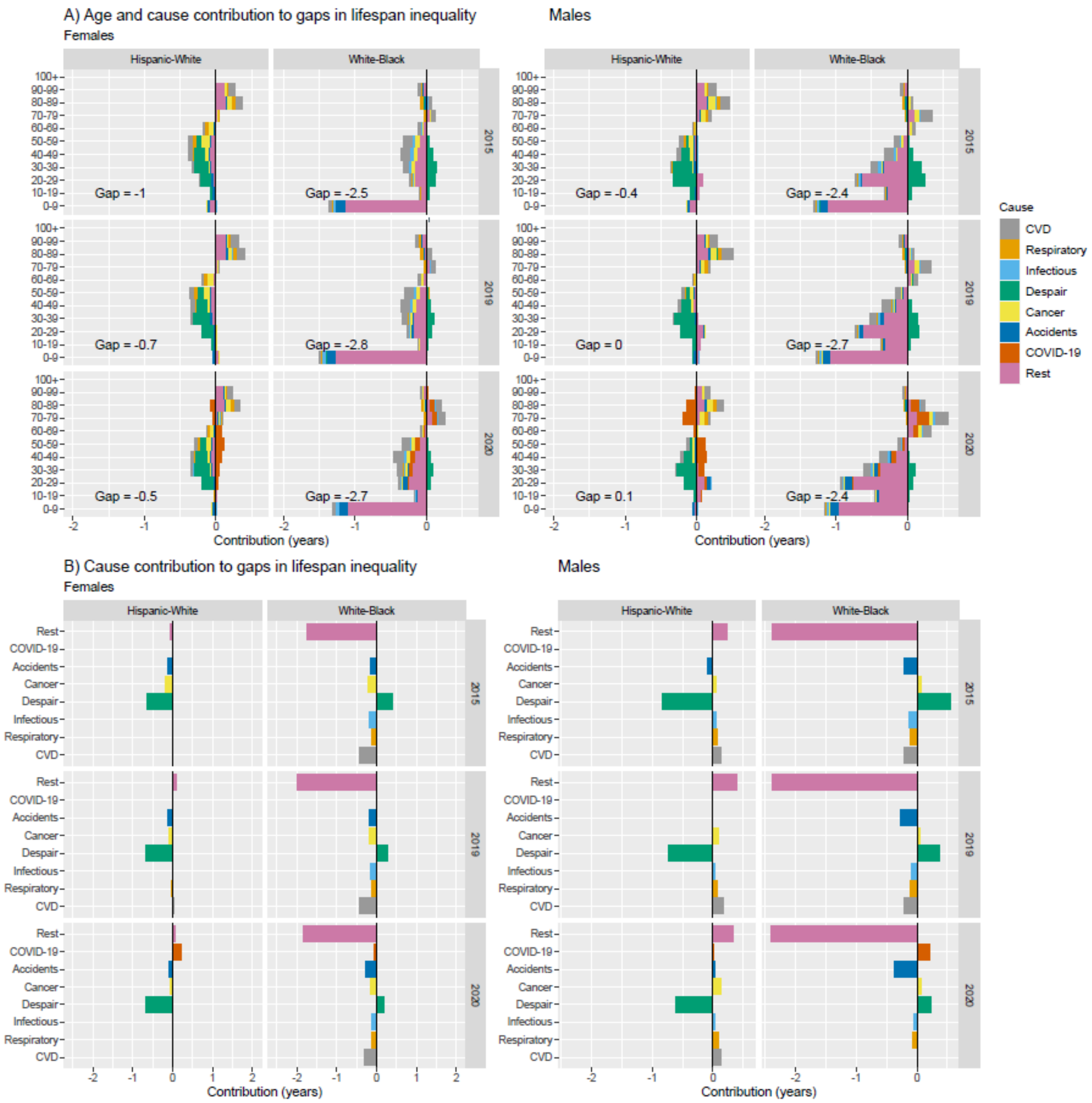

Supplement: Supplementary File [file pnas.2205813119.sapp.pdf]
